# Gan-Lu-Yin (Kanroin), Traditional Chinese Herbal Extracts, Reduces Osteoclast Differentiation In Vitro and Prevents Alveolar Bone Resorption in Rat Experimental Periodontitis

**DOI:** 10.3390/jcm10030386

**Published:** 2021-01-20

**Authors:** Yuji Inagaki, Jun-ichi Kido, Yasufumi Nishikawa, Rie Kido, Eijiro Sakamoto, Mika Bando, Koji Naruishi, Toshihiko Nagata, Hiromichi Yumoto

**Affiliations:** Department of Periodontology and Endodontology, Institute of Biomedical Sciences, Tokushima University Graduate School, Tokushima 770-8504, Japan; kido.jun-ichi@tokushima-u.ac.jp (J.K.); nishikawa.yasufumi@tokushima-u.ac.jp (Y.N.); rie.kido@tokushima-u.ac.jp (R.K.); sakamoji@tokushima-u.ac.jp (E.S.); banchi@tokushima-u.ac.jp (M.B.); naruishi@tokushima-u.ac.jp (K.N.); nagata.toshihiko@tokushima-u.ac.jp (T.N.); yumoto@tokushima-u.ac.jp (H.Y.)

**Keywords:** Gan-Lu-Yin, periodontitis, herbal medicine, osteoclastogenesis

## Abstract

Gan-Lu-Yin (GLY), a traditional Chinese herbal medicine, shows therapeutic effects on periodontitis, but that mechanism is not well known. This study aims to clarify the precise mechanism by investigating the inhibitory effects of GLY extracts on osteoclastogenesis in vitro and on bone resorption in periodontitis in vivo. RAW264.7 cells are cultured with soluble receptor activator of nuclear factor-kappa B (sRANKL) and GLY extracts (0.01–1.0 mg/mL), and stained for tartrate-resistant acid phosphatase (TRAP) to evaluate osteoclast differentiation. Experimental periodontitis is induced by placing a nylon ligature around the second maxillary molar in rats, and rats are administered GLY extracts (60 mg/kg) daily for 20 days. Their maxillae are collected on day 4 and 20, and the levels of alveolar bone resorption and osteoclast differentiation are estimated using micro-computed tomography (CT) and histological analysis, respectively. In RAW264.7 cells, GLY extracts significantly inhibit sRANKL-induced osteoclast differentiation at a concentration of more than 0.05 mg/mL. In experimental periodontitis, administering GLY extracts significantly decreases the number of TRAP-positive osteoclasts in the alveolar bone on day 4, and significantly inhibits the ligature-induced bone resorption on day 20. These results show that GLY extracts suppress bone resorption by inhibiting osteoclast differentiation in experimental periodontitis, suggesting that GLY extracts are potentially useful for oral care in periodontitis.

## 1. Introduction

Periodontal diseases are caused by periodontopathic bacteria in dental plaque, occlusal trauma, and several systemic diseases, and induced inflammation in periodontal tissues and resorption of alveolar bone. Periodontal diseases are strongly associated with various systemic inflammatory diseases, such as rheumatoid arthritis (RA), diabetes, and cardiovascular diseases, including coronary heart disease (CHD) and stroke. Recently, it has been reported that periodontal diseases stimulate the immune systems not only in periodontal lesions, but also in various systemic tissues [1,2]. Because periodontal disease affects the expression of biomarkers, including cytokines, oxidative stress markers, receptors, and immunoglobulins, not only in periodontal tissues, but also in saliva, serum, and systemic tissues, many biomarkers for periodontitis has been systemically researched to date and recently expected to be a valuable prognostic biomarker of periodontitis and systematically inflammatory diseases.

Pro-inflammatory cytokines, such as tumor necrosis factor (TNF-α) and interlukin (IL-6) directly, and indirectly activated osteoclast differentiation through receptor activator of nuclear factor-κB ligand (RANKL)/RANK pathway and induced alveolar bone resorption in periodontitis [3]. The pathological bone resorption is caused by stimulating osteoclast differentiation and formation, and this process is regulated by some enzymes and transcription factors, including cathepsin K, nuclear factor of activated T-cells cytoplasmic 1 (NFATc1), and dendritic cell-specific transmembrane protein (DC-STAMP) [3,4,5,6,7,8]. TNF-α antagonists, inhibitors of the IL-1 receptor, IL-6 receptor and RANKL/RNAK interaction, and cathepsin K inhibitor are thought to inhibit alveolar bone resorption in periodontitis [3]; however, these agents have not been used as an internal medication to prevent alveolar bone resorption in periodontitis. Seto et al. [9] and Tokunaga et al. [10] reported that simvastatin and parathyroid hormone-stimulated differentiation of rat calvarial cells and recovered the ligature-induced alveolar bone resorption in rat experimental periodontitis. Furthermore, calcitonin significantly decreased the numbers of inflammatory cells and tartrate-resistant acid phosphatase (TRAP)-positive cells (osteoclasts) and inhibited the ligature-induced alveolar bone resorption in experimental periodontitis [11]. These agents and hormones showed the suppression of alveolar bone resorption when they were locally injected into rat periodontal tissues.

Some medical plant extracts and herbal medicines show therapeutic effects against periodontal diseases with inflammation and alveolar bone resorption [12,13,14]. Harmine, a β-carboline alkaloid from *Peganum harmala*, inhibited NFATc1 expression and RANKL-induced osteoclast formation [15], and alisol-B, a phyto-steroid from *Alisma orientale* Juzepczuk, inhibited RANKL/RANK signaling, NFATc1 expression and osteoclast formation [16], and glymnasterkoreayne F from *Gymnaster koraiensis* suppressed NFATc1 expression, decreased the levels of cathepsin K and TRAP and inhibited osteoclast differentiation from bone marrow-derived macrophages [17]. On the other hand, the traditional Chinese and Japanese medicines contain multiple components derived from herbs and natural plant extracts, have pharmacological effects with anti-inflammatory, anti-oxidant, and anti-microbial characteristics, etc., for treatment of periodontal diseases [18,19]. Shi-Quan-Da-Bu-Tang (Juzentaihoto), a medicine that contains 10 herbs, showed antibacterial activity against *Porphyromonas gingivalis* and reduced *P. gingivalis*-induced alveolar bone resorption by inhibiting osteoclast differentiation in rat experimental periodontitis [20]. Da-Huang-Gan-Cao-Tang (Daiokanzoto), a crude extracts of Rhubarb rhizomes and Glycyrrhiza roots, was thought to suppress bone resorption in periodontitis, since this medicine inhibited *P. gingivalis*-induced nuclear factor (NF-κB) activity, IL-6 expression and matrix metalloproteinase (MMP)-1 activity in human gingival epithelial cells and fibroblasts [19,21].

In the clinical stage, Gan-Lu-Yin (GLY) formula (Kanroin) is approved as a medicine to treat oral inflammations, such as periodontitis, stomatitis, and glossodynia, by the Japanese Ministry, Labor and Welfare, and contains nine herbs, including Artemisia Capillaris flower (*Artemisia capillaris* Thunberg), Scutellaria root (*Scutellaria baicalensis* Georgi), Loquat leaf (*Eriobotrya japonica* Lindley), Immature orange (*Citrus aurantium* Linné var. *daidai* Makino), Rehmannia root (*Rehmannia glutinosa* Liboschitz var. *purpurea* Makino), Asparagus root (*Asparagus cochinchinensis* Merrill), Ophiopogon root (*Ophiopogon japonicus* Ker-Gawler), Dendrobium (*Dendrobium nobile* Lindley), and Glycyrrhiza (*Glycyrrhiza uralensis* Fisher) [22,23,24]. GLY extracts suppressed the migration of vascular smooth muscle cells (VSMCs) by inhibiting MMP-2 and -9 [24] and inhibited vascular endothelial growth factor (VEGF) expression and tube formation in human umbilical vein endothelial cells (HUVEC) [25], and further suppressed TNF-α expression in human oral cancer cells through ERK and NF-κB pathway [22], suggesting that GLY extracts have an anti-inflammatory reaction and inhibitory action of tissue destruction. Although GLY extracts have been used for medical treatments of periodontitis, the effect of GLY extracts on alveolar bone metabolism, and their detailed mechanism are not well known. In the present study, to investigate the possibility of GLY extracts as the therapeutic agent for periodontitis, we examined the inhibitory effects of GLY extracts on the differentiation of pre-osteoclastic cells using a murine osteoclast precursor cells treated with sRANKL in vitro. Further, we assessed the preventive effect of GLY extracts on alveolar bone resorption in rat experimental periodontitis using micro-computed tomography (CT) and histological sections in vivo.

## 2. Experimental Section

### 2.1. Reagents

The powder of GLY extracts was supplied by Sunstar (Osaka, Japan). Briefly, the powder was prepared by extracting and drying a mixture of Artemisia Capillaris flower, Scutellaria root, Loquat leaf, Immature orange, Rehmannia root, Asparagus root, Ophiopogon root, Dendrobium, and Glycyrrhiza. Each herb were equally weighted (12.5 g) and extracted with boiling in 125 mL of distilled water; then, the extract was filtered and dried by heating under reduced pressure. Alpha-modified Eagle’s minimal essential medium (α-MEM) and TRAP staining kit were purchased from Wako (catalog number 135-15175 and 294-67001, Osaka, Japan). Recombinant murine sRANKL was from Peprotech (catalog number 315-11, Rocky Hill, NJ, USA), and rabbit polyclonal antibodies against cathepsin K, NFATc1, ephrin B2, and mouse monoclonal antibody against β-actin were from Abcam (catalog number ab19027, ab25916, ab150411 and ab49900, Cambridge, UK). Mouse monoclonal antibody against DC-STAMP was from EMD Millipore (catalog number MABF39-I, Temecula, CA, USA). Horseradish peroxidase (HRP)-conjugated goat anti-rabbit IgG antibody, HRP-conjugated goat anti-rat IgG antibody, and HRP-conjugated horse anti-mouse IgG antibody were from Cell Signaling Technology (catalog number 7074, 7077, and 7076, Beverly, MA, USA).

### 2.2. Cell Culture and Osteoclastogenesis

The murine macrophage cell line, RAW264.7, was obtained from the American Type Culture Collection (ATCC, Rockville, MD, USA), and were seeded in 6-wells or 12-wells plates (IWAKI, Chiba, Japan) at 10,000 cells per cm^2^ and cultured in α-MEM supplemented with 10% fetal bovine serum (FBS) at 37 °C in a humidified atmosphere of 5% CO_2_ and 95% air. RAW264.7 cells were differentiated to osteoclasts by sRANKL (50 ng/mL) according to the method of Zhang et al. [26]. To examine the effect of GLY extracts on osteoclast differentiation, the cells were cultured with sRANKL and GLY extracts (0.01–1.0 mg/mL) for five days and stained with the TRAP staining kit according to the manufacturer’s instructions. TRAP-positive multinucleated cells (MNCs) with more than three nuclei were counted under a phase contrast microscope with x100 magnification, and its number was counted by an independent examiner in a blind manner. The significantly differences were calculated with the number of TRAP-positive MNCs of sRANKL alone treatment as a control. These procedures were repeated three times by using triplicate RAW264.7 cell cultures.

### 2.3. Cell Viability Assay

Cell viability was examined using a Cell Counting Kit-8 (CCK-8; catalog number CK04, Dojindo, Kumamoto, Japan) according to the manufacturer’s instructions. Briefly, RAW264.7 cells were seeded at 5000 cells/well in a 96-well plate (SUMITOMO BAKELITE, Tokyo, Japan) and cultured in a growth medium (α-MEM–10% FBS). After 48 h, cells were exposed with medium alone or 0.01–1.0 mg/mL GLY extracts for 48 h, and then incubated with CCK-8 solution. After the incubation of cells with CCK-8 solution for 1 h, the absorbance of each well was measured at 450 nm using a microplate reader (iMark^™^; Bio-Rad, Hercules, CA, USA). The percentages of cell viability were calculated according to the values of the medium alone as 100%. These procedures were repeated two times by using triplicate RAW264.7 cell cultures.

### 2.4. Western Blot Analysis

RAW264.7 cells differentiated to osteoclasts by sRANKL were cultured with GLY extracts (0.05–0.5 mg/mL) in 6-wells plates for five days. The cells were lysed in 50 µL of RIPA lysis buffer (catalog number sc-24948, Santa Cruz Biotechnology Inc., Santa Cruz, CA, USA). Equal amounts of proteins (30 µg) were denatured and separated by 10% sodium dodecyl sulfate polyacrylamide gel electrophoresis (SDS-PAGE) and transferred to nitrocellulose membranes (Bio-Rad). After blocking the membranes with 5% non-fat dry milk in 50 mM Tris-buffered saline (pH 7.6) containing 0.05% Tween 20, proteins on the membrane were reacted with optimally diluted primary antibodies of β-actin, cathepsin K, DC-STAMP, ephrin B2, and NFATc1 overnight at 4 °C, and then incubated with each HRP-conjugated secondary antibodies (1/2000 dilution) at room temperature for 1 h. After washing a membrane, the blots were developed by Amercham ECL Western Blotting Detection Reagents and visualized using Image Quant LAS 500 (GE Healthcare, Little Ckalfont, UK). These procedures were repeated 2–3 times.

### 2.5. Animals

Experiments using animal was approved by the Animal Research Control Committee of the Tokushima University Graduate School (T28-90). The study complied with the Animal Research: Reporting in vivo Experiments guidelines developed by the National Centre for the Replacement, Refinement, and Reduction of Animals in Research (NC3Rs). Forty-two male 8-week-old Wistar rats (220–230 g, Charles River Laboratories Japan, Inc., Yokohama, Japan) were housed in a temperature- and humidity-controlled room (23 ± 1 °C and 60 ± 5% relative humidity) with 12 h light/dark cycle and fed with standard rodent chow and water ad libitum.

### 2.6. Experimental Periodontitis

Twenty-eight rats were randomly divided into two groups as follows; (1) a GLY administration group in which rats were orally administered with GLY extracts (60 mg/kg) for 20 days from the day of ligaturing (*n* = 18) and (2) a non-administration group in which rats were orally-administered with the same volume of distilled water for 20 days as control (*n* = 10). The number of rats was justified using size calculation based on the previous report [27]. However, considering the possibility that the rats could be lost by administering GLY extracts during the experimental period, a sample size of 18 rats was planned in the GLY administration group compared to 10 rats in the control group. Experimental periodontitis of rats was induced according to the previous method [28,29,30,31]. Briefly, the cervical area of the right second molar of the rat maxilla was ligatured with nylon thread (No. 5-0; Natsume Corporation, Tokyo, Japan) under anesthesia with sodium pentobarbital (day 0). The ligature was knotted on the buccomesial to confirm the ligature remained during the experimental period, and the ligature was checked every day to ensure subgingival placement. The left second molar of rat maxilla was used as the non-ligatured control.

### 2.7. Microcomputed Tomography (µCT) Analysis of Alveolar Bone

Eighteen rats, which consist of twelve rats of the GLY administration group and six rats of control, were sacrificed after a measurement of body weight on day 20, and their maxillae and peripheral blood were collected. The specimens of alveolar bone were immediately fixed in 10% neutral-buffered formalin, and alveolar bone resorption levels were analyzed using micro-CT system (SkyScan 1176, Bruker, Billerica, MA, US) according to the previous method [28,29]. The distance from the buccal cement-enamel junction (CEJ) to the alveolar bone crest (ABC) of the second molar was measured in the frontal section as a marker of bone height. To ensure reproducibility of the alignment of the micro-CT image, the buccal cusp tip of the second molar was placed such that they superimposed on the corresponding palatal cusp tip. The distance between CEJ and ABC was measured at four points of the buccal side of the second molar, including mesial, distal, and middle (furcation) sites, and its average was calculated.

### 2.8. Histological Analysis

Ten rats, which consist of six rats of the GLY administration group and four rats of control, were sacrificed on day 4 after ligature placement, and their maxillae were dissected. The specimens of alveolar bone were immediately fixed in 10% neutral-buffered formalin and decalcified with 10% EDTA for 21 days, and embedded in paraffin (Paraplust Plus, Sigma, St Louis, MO). Histological sections with alveolar bone were longitudinally prepared at 6 µm-thick widths and stained with the TRAP staining kit according to the manufacturer’s instructions. The TRAP-positive MNCs in the stained section were observed as osteoclasts using an optical microscope (Microphoto V series, Nikon, Tokyo, Japan), and the TRAP-positive MNCs were defined by cells with more than three nuclei under a light microscope and counted according to a previous report [11]. Briefly, the number of TRAP-positive MNCs in the alveolar bone was measured in the square of 450 × 600 µm microscopic visual fields at a magnification of x200, using 16 sections from four control rats (non-ligatured left side, *n* = 8; ligatured right side, *n* = 8), and 24 sections from six GLY administration rats (non-ligatured left side, *n* = 12; ligatured right side, *n* = 12). The same visual fields of every second molar were analyzed by an independent examiner in a blind manner using a light microscope and digital camera and averaged the number of osteoclasts in buccal and palatal sides.

### 2.9. Analysis of Bone Metabolism Marker in Rat Serum

Rats were administered GLY extracts or distilled water for 20 days, and their 5mL of peripheral blood was collected to determine levels of bone metabolism marker. Sera were prepared from the blood samples, and the amounts of NTx-1 (cross-linked N-telopeptide of collagen type I) and osteocalcin in sera were determined using each ELISA kit (catalog number LS-F21857 and LS-F22801, Life Span BioSciences, Inc., Seattle, WA, US) according to the manufacturer’s instructions.

### 2.10. Analysis of Systemic Bone Volume Fraction and Bone Mineral Density

Fourteen rats were randomly divided into the GLY administration group and the non-administration group as control (*n* = 7/group). The administration group was orally-administered with GLY extracts (60 mg/kg) every day, and the non-administration group was administered with the same volume of distilled water every day. On day 28, the rats’ bodyweights were measured, and the rats were sacrificed, followed by the collection of femurs. The femurs were immediately fixed in 70 % ethanol, and then bone volume fraction (BV/TV: bone volume/tissue volume), bone mineral contents (BMC), and bone mineral density (BMD) were analyzed by Kureha Special Laboratory (Fukushima, Japan).

### 2.11. Statistical Analysis

Statistical analyses were performed with SPSS Statistics version 20 (Armonk, NY, USA). After a Anderson–Darling test, comparisons between two experimental groups were performed by Student’s *t*-test, and those among three groups or more were analyzed using one-way ANOVA followed by a post-hoc Tukey-Kramer test. In all statistical analyses, *p* values of less than 0.05 were considered significant.

## 3. Results

### 3.1. Effect of GLY Extracts on Cell Proliferation of RAW264.7 Cells

**Figure 1:**
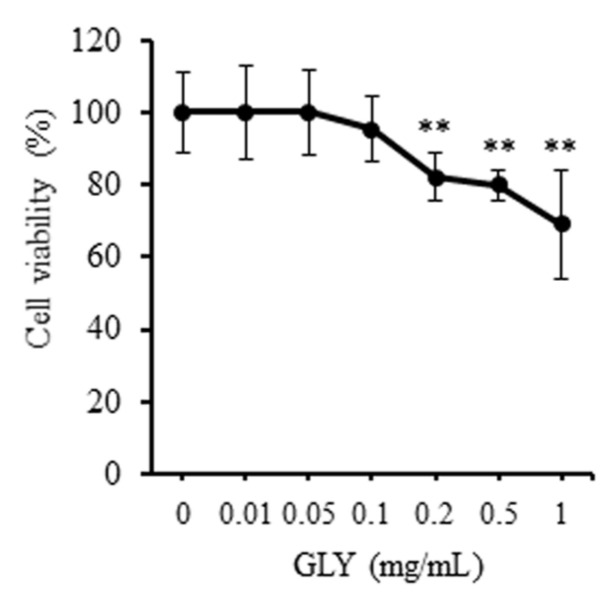
Effects of Gan-Lu-Yin (GLY) extracts on cell viability in RAW264.7 cells. The cells were seeded at 5000 cells/well in a 96-well plate. After sub-confluency, the cells were incubated in the absence or presence of various concentrations (0.01, 0.05, 0.1, 0.2, 0.5, and 1.0 mg/mL) of GLY extracts. The cell viability was assessed for the cultivation of 48 h using Cell Counting Kit-8. The percentages of cell viability were calculated according to the values of the absence of GLY extracts as 100%. Data are presented as the mean ± SD. ** *p* < 0.01 compared with the cultivation with 10% fetal bovine serum (FBS) alone. These procedures were repeated two times by using triplicate RAW264.7 cell cultures.

The inhibitory effect of GLY extracts on the proliferation of RAW264.7 cells was investigated at 48 h-culture. 0.01–0.1 mg/mL of GLY extracts did not significantly influence the cell viability of RAW264.7 cells (Figure 1). However, GLY extracts at a high concentration of 0.2–1.0 mg/mL significantly inhibited the cell viability of RAW264.7 cells and decreased its viability to approximately 20–30% compared to control (GLY: 0 mg/mL).

### 3.2. GLY Extracts Inhibit Osteoclast Differentiation

**Figure 2:**
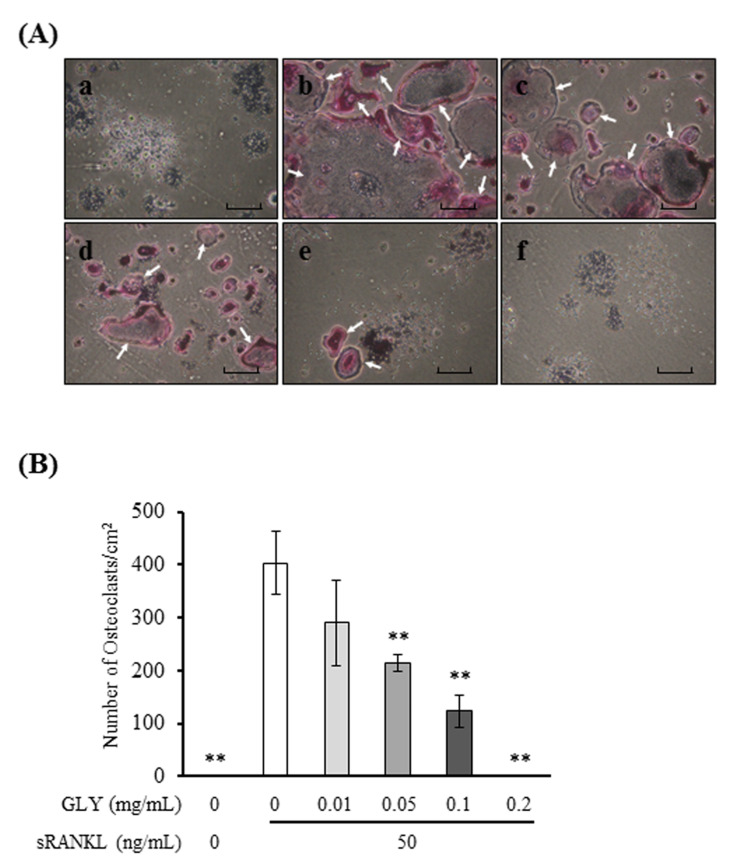
Effects of GLY extracts on the receptor activator of nuclear factor-κB ligand (RANKL)-induced osteoclast differentiation. (**A**) Microscopic observations for tartrate-resistant acid phosphatase (TRAP) staining of osteoclast-like positive multinucleated cells (MNCs) derived from soluble RANKL (sRANKL)-treated RAW264.7 cells in the absence or presence of various concentrations of the GLY extracts for five days. (**a**) no-treated with sRANKL and absences of GLY extracts; (**b**) absence of GLY extracts; (**c**) 0.01 mg/mL of GLY extracts; (**d**) 0.05 mg/mL of GLY extracts; (**e**) 0.1 mg/mL of GLY extracts; (**f**) 0.2 mg/mL of GLY extracts. Magnification x100, the arrowheads indicate TRAP-positive osteoclasts. Scale bars represent 300 µm. (**B**) Quantitative analysis of TRAP-positive MNCs with three or more nuclei in each well. Data are presented as the mean ± SD. ** *p* < 0.01 compared with the sRANKL treatment in the absence of GLY extracts. These procedures were repeated three times by using triplicate RAW264.7 cell cultures.

To investigate the effects of GLY extracts on osteoclast differentiation, RAW264.7 cells were cultured with sRANKL and GLY extracts. When RAW264.7 cells were stimulated with sRANKL (50 ng/mL), the cells were differentiated to TRAP-positive MNCs, osteoclasts. GLY extracts inhibited sRANKL-induced osteoclast differentiation and decreased the number and size of osteoclasts (Figure 2A), and significantly decreased the number of osteoclasts in a dose-dependent manner (0.05–0.2 mg/mL) (Figure 2B). GLY extracts at high concentration (0.5 and 1.0 mg/mL) completely suppressed sRANKL-induced osteoclast differentiation (Figure S1). These results showed that GLY extracts at a concentration of more than 0.05 mg/mL inhibited osteoclast differentiation in vitro.

### 3.3. GLY Extracts Inhibited the Expressions of Osteoclast Markers in RAW264.7 Cells

**Figure 3:**
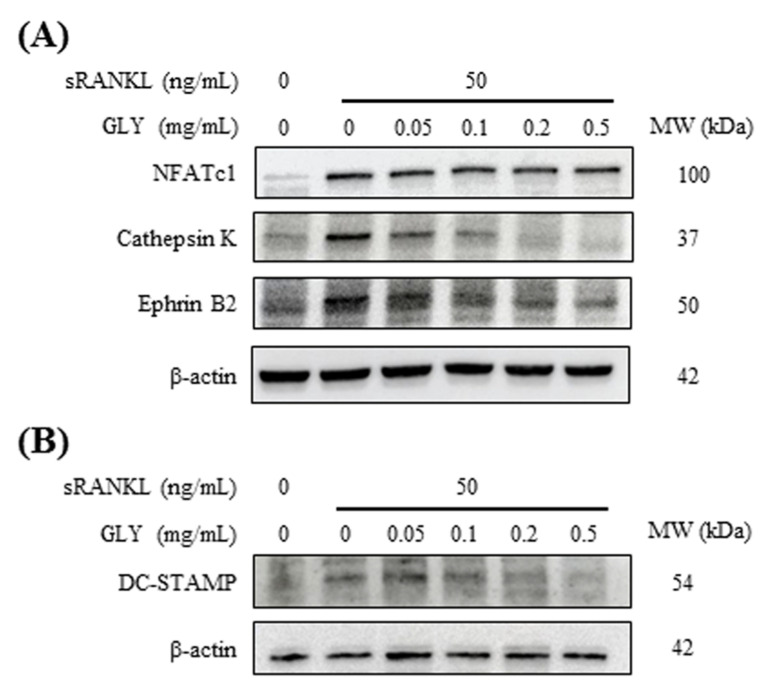
Effects of GLY extracts on the expression of osteoclast-specific markers in RAW264.7 cells. (**A**) sRANKL-treated RAW264.7 cells were incubated in the absence or presence of various concentrations (0.05, 0.1, 0.2 and 0.5 mg/mL) of GLY extracts for five days. The expression of NFATc1, cathepsin K and ephrin B2 in the cells were evaluated using Western blot analysis. Β-actin served as a loading control. (**B**) The expression of DC-STAMP and β-actin in the cells were evaluated using Western blot analysis.

sRANKL (50 ng/mL) increased the expression of osteoclast markers, including NFATc1, cathepsin K, ephrin B2 and DC-STAMP in RAW264.7 cells (Figure 3). When sRANKL-induced osteoclastic cells were cultured with GLY extracts, GLY extracts inhibited the expression of cathepsin K, ephrin B2 and DC-STAMP in a dose-dependent manner (Figure 3A,B; 0.05–0.5 mg/mL). In contrast, NFATc1 protein level in sRANKL-induced osteoclastic cells was not influenced by GLY extracts at 0.05–0.5 mg/mL concentration.

### 3.4. GLY Extracts Inhibited Alveolar Bone Resorption in Rat Experimental Periodontitis

**Figure 4:**
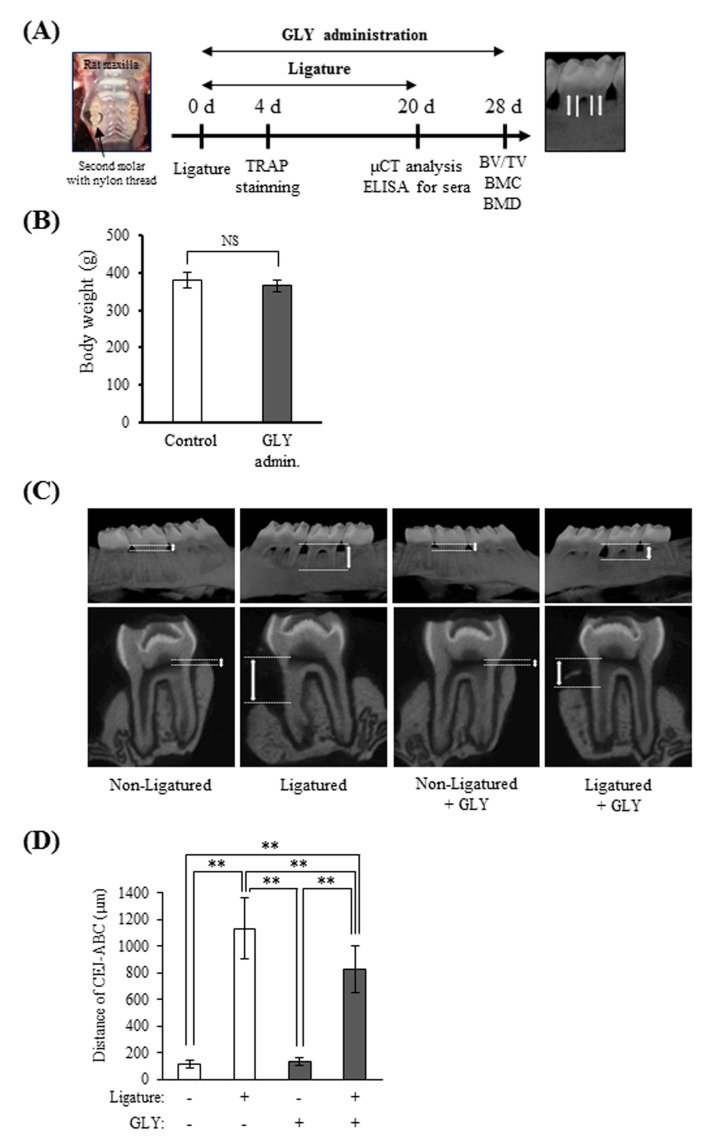
The effects of GLY extracts on bone resorption in ligature-induced periodontitis. (**A**) Experimental design and the points of linear measurement of the cement-enamel junction (CEJ) to the alveolar bone crest (ABC) by micro-CT analysis. (**B**) Body weights of rats on day 20 from the placement of ligature. Control; group of oral administration of distilled water for 20 days (*n* = 6), GLY admin.; group of oral administration with 60 mg/kg of GLY extracts for 20 days (*n* = 12). (**C**) Representative micro-CT images of the frontal and sagittal sections of maxillary secondary molars from rats. (**D**) Distance from the CEJ to the ABC as a marker of alveolar bone resorption in micro-CT. Data are presented as the mean ± SD from six or twelve rats per group. Double asterisk indicates *p* < 0.01. NS indicates no significant differences.

The effect of GLY extracts on alveolar bone resorption was evaluated using rats with ligature-induced periodontitis (Figure 4A). There were no significant differences in body weight of rats between the administration group and non-administration group on day 20 of ligature placement, and the mean of rat body weight was 365 ± 16 g and 380 ± 20 g, respectively (Figure 4B). In µCT images of alveolar bone of rats with periodontitis, bone resorption was observed around the ligatured second molar (right side) as compared with the non-ligatured side (left side) (Figure 4C). Although the length between cement-enamel junction (CEJ) and alveolar bone crest (ABC) was 116.7 ± 31.3 µm in the non-ligatured control group, bone resorption in the ligatured control group remarkably increased, and its length was 1134.7 ± 231.2 µm. When GLY extracts were administered to rats, the length in the ligatured GLY administration group was 829.6 ± 173.6 µm in spite of the length in the non-ligatured GLY administration group was 134.8 ± 28.5 µm (Figure 4D). The administration of GLY extracts decreased the level of bone resorption of the ligatured control without GLY extracts by approximately 27%, suggesting that GLY extracts significantly inhibited the ligature-induced bone resorption.

### 3.5. GLY Extracts Inhibited Osteoclast Differentiation in Rat Experimental Periodontitis

**Figure 5:**
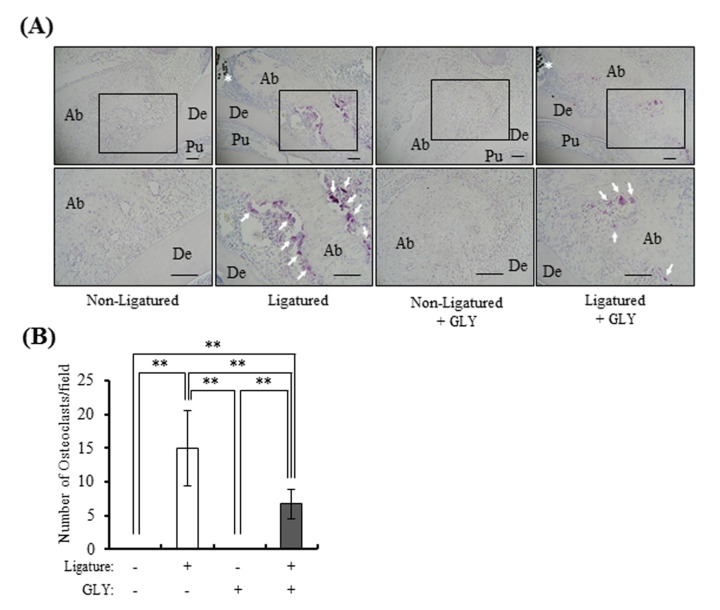
The effect of GLY extracts on osteoclast differentiation in ligature-induced periodontitis. Microscopic observations were performed on the decalcified paraffin sections with TRAP staining on day 4 from the placement of ligature. (**A**) Representative images have shown TRAP-positive osteoclasts in the alveolar bone around maxillary secondary molar in the non-ligatured and ligatured buccal side; upper panels: x100 magnification, lower panels: x200 magnification. Ab, alveolar bone; De, dentin; Pu, pulp; the asterisk indicates the nylon ligature, and the arrowheads indicate TRAP-positive osteoclasts, Scale bars represent 100 µm. (**B**) Quantitative analysis of TRAP-positive osteoclasts on day 4 in the ligatured buccal and palatal side. TRAP-positive cells per visual field, counted 16–24 visual fields. Data are presented as the mean ± SD from four or six rats per group. Double asterisk indicates *p* < 0.01. Control; group of oral administration of distilled water for four days (*n* = 4), GLY admin.; group of oral administration with 60 mg/kg of GLY extracts for four days (*n* = 6).

The effect of GLY extracts on osteoclast differentiation was histologically investigated at ligatured buccal and palatal sides around rat maxillary secondary molar. The TRAP-positive MNCs, osteoclasts, were not observed in the non-ligatured control group and non-ligatured GLY administration group, but the numerous osteoclasts were observed in the ligatured control group (Figure 5A and Figure S2). The number of osteoclasts were suppressed by GLY extracts, and the inhibitory rates of osteoclast differentiation were approximately 55% at ligatured control group (Figure 5B).

### 3.6. GLY Extracts Decreased Serum NTx-1 Level in Rat Experimental Periodontitis

**Figure 6:**
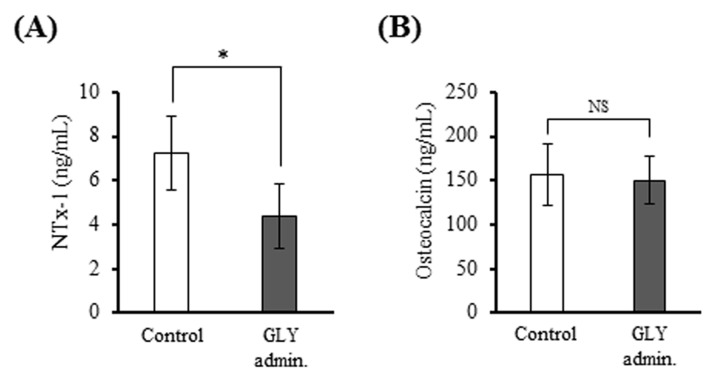
Comparison of the number of bone biomarkers in sera of ligature-induced periodontitis. (**A**) Serum concentrations of NTx-1 as a bone resorption marker. (**B**) Serum concentrations of osteocalcin as a bone formation marker. Data are presented as the mean ± SD from six or twelve rats per group. * *p* < 0.05 compared with control. NS indicates no significant differences. Control; group of administration of distilled water for 20 days (*n* = 6), GLY admin.; group of oral administration with 60 mg/kg of GLY extracts for 20 days (*n* = 12).

The level of NTx-1, bone resorption marker, in sera from the GLY administration group (4.4 ± 1.4 ng/mL) were significantly lower than that of the non-administration group (7.2 ± 1.7 ng/mL) (Figure 6A), whereas there was no significant difference in the level of osteocalcin, osteoblastic cell marker, between two groups (Figure 6B).

### 3.7. GLY Extracts Not Affect Bone Structure and Bone Mineral Density in Rat with Experimental Periodontitis

**Figure 7:**
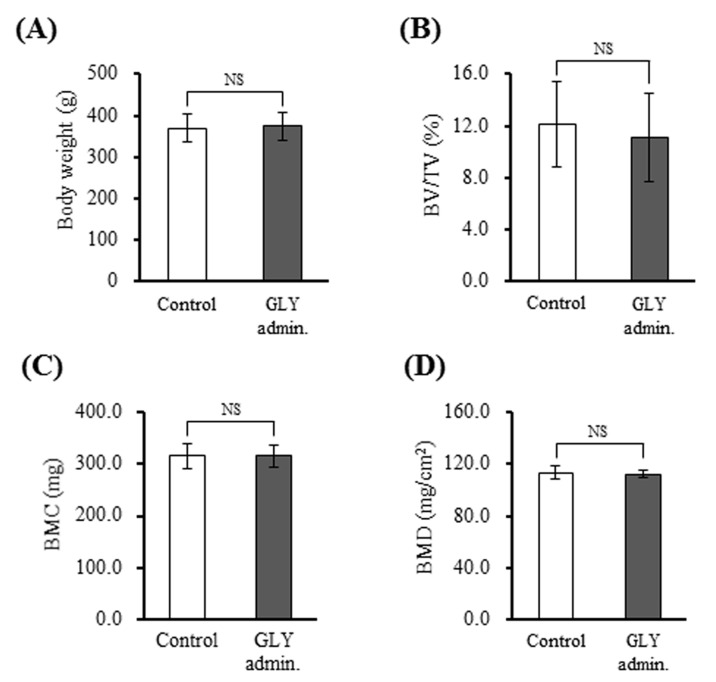
Effects of GLY extracts on systemic bone metabolism. (**A**) Body weights of rats, (**B**) Bone volume fraction (BV/TV, %), (**C**) Bone mineral contents (BMC, mg), (**D**) Bone mineral density (BMD, mg/cm^2^). Data are presented as the mean ± SD from seven rats per group. NS indicates no significant differences. Control; group of administration of distilled water for 28 days (*n* = 7), GLY admin.; group of oral administration with 60 mg/kg GLY extracts for 28 days (*n* = 7).

To investigate the effects of GLY extracts on systemic bone metabolism, BV/TV, BMC, and BMD were assessed using the rat femurs. Figure 7A shows no differences in the body weight between the GLY administration group (384 ± 26 g) and control (382 ± 12 g). BV/TV of the GLY administration group and control were 12.1 ± 2.5 and 13.2 ± 2.1 %, respectively (Figure 7B). BMC of the GLY administration group and control were 322.9 ± 12.2 mg and 318.9 ± 24.3 mg, respectively (Figure 7C). Further, BMD of the GLY administration group and control were 113.1 ± 2.2 mg/cm^2^ and 114.9 ± 2.5 mg/cm^2^, respectively (Figure 7D). These µCT and DXA (Dual energy X-ray Absorptiometry) assessments indicated that no differences in BV/TV, BMC, and BMD between the GLY administration group and the non-administration control group. Thus, in this experimental period, GLY extracts did not systemically affect bone metabolism in vivo.

## 4. Discussion

The present in vitro study using RAW264.7 cells showed that GLY extracts significantly inhibited sRANKL-induced osteoclast differentiation and decreased the expressions of osteoclast differentiation markers, such as cathepsin K, ephrin B2, and DC-STAMP. Further, the present in vivo study showed that administering GLY extracts for rat experimental periodontitis inhibited the ligature-induced alveolar bone resorption and significantly decreased the number of TRAP-positive osteoclasts around a ligature in the alveolar bone. GLY formula consists of nine herbs and contains 14 main components with pharmacological effects, including baicalin, baicalein, oroxylin A-7-O-glucuronide, wogonin, oroxylin A, naringin, neohesperidin, liquiritigenin, liquiritin and glycyrrhizic acid [32]. GLY extracts have anti-inflammatory and anti-tumor effects, which suppress TNF-α level in human oral carcinoma cells [22], angiogenesis in HUVEC [25], and migration of VSMCs [24]. Regarding the effective concentration, GLY extracts showed an inhibition of TNF-α level in oral carcinoma cells at more than 0.2 mg/mL, cell migration, and MMP-9 production in VSMCs at more than 0.125 mg/mL, and angiogenesis of HUVEC at 1.0–1.5 mg/mL [22,24,25]. In contrast, in osteoclast precursor (RAW264.7 cells), GLY extracts exhibited significant inhibitory effects on osteoclast differentiation, the production of cathepsin K, and ephrin B2 at comparatively low concentration, such as 0.05 and 0.1 mg/mL (Figure 2 and Figure 3). Moreover, in the present study, GLY extracts at more than 0.2 mg/mL significantly attenuated the cell proliferation (Figure 1). However, several studies indicated that GLY extracts inhibit cell viability at 0.25 mg/mL on murine leukemia cells, 0.5 mg/mL on VSMCs and 1.5 mg/mL on HUVEC, respectively [23,24,25]. Our results suggested that osteoclasts may have a high sensitivity for GLY extracts.

On the other hand, Baicalin, at a high concentration of 200 mg/kg, suppressed the ligature-induced alveolar bone resorption, and slightly increased the area of collagen fibers in rat gingival connective tissues when that area of gingival tissues in the ligatured group was compared [33]. Glycyrrhizic acid and Naringin inhibited RANKL-induced osteoclast differentiation in murine bone marrow macrophages (BMMs), and preserved bone mass and trabecular structure in proximal tibial trabecular of ovariectomized mice, and suppressed bone resorption in pit assay [34,35]. Furthermore, Wogonin inhibited LPS-induced osteoclast formation in co-cultures with mouse calvaria osteoblasts and BMMs and suppressed lipopolysaccharide (LPS)-induced RANKL expression and recovered LPS-decreased osteoprotegerin production in osteoblasts [36]. In the present study, GLY extracts suppressed osteoclast differentiation in vitro and bone resorption in vivo, and these effects were similar to the effects of Bacicalin, Glycyrrhizic acid, Naringin, and Wogonin on bone metabolism, suggesting that multiple components in GLY extracts influence the inhibition of alveolar bone resorption. Wogonin contained in GLY extracts influenced osteoblasts, as well as osteoclasts [36]. We did not investigate the direct effect of GLY extracts on osteoblasts in the present study, but an administration of GLY extracts to rats did not change osteocalcin level in rat serum. Since osteocalcin is an indicator of bone formation activity in osteoblasts [37], the reason for the different effect on osteoblasts between GLY extracts and wogonin is not known. However, there may be differences between in vitro and in vivo, or the effects derived from other components in GLY extracts.

Regarding the inhibitory effects of GLY extracts on osteoclast differentiation, GLY extracts suppressed RANKL-induced cathepsin K, DC-STAMP, and ephrin B2 productions in RAW264.7 cells (Figure 3). Cathepsin K, a member of the papain family of cysteine proteases, is highly expressed in osteoclasts and plays an important role in bone resorption by degrading type I collagen and osteopontin in bone tissues [4,38]. DC-STAMP is essential for cell-cell fusion of mononuclear pre-osteoclasts and to mature osteoclasts with multi-nuclear [5,6,7]. Cathepsin K and DC-STAMP are thought as osteoclastic specific markers to control pathological bone resorption in osteogenic diseases because they are closely related to osteoclast differentiation and function. Glycyrrhizin (glycyrrhizic acid) and Naringin contained in GLY extracts also suppressed the expressions of cathepsin K and DC-STAMP mRNAs, and then abrogated osteoclast formation in BMMs [35,39]. The inhibitory effect of GLY extracts on cathepsin K and DC-STAMP expression may be dependent on the pharmacological effects of Glycyrrhizin and Naringin. In contrast, Glycyrrhizin inhibited the expression of NFATc1, which is a member of the NFAT transcription factor family and a key regulator of RANKL-induced osteoclast differentiation [8]. Baicalein also suppressed NFATc1 expression and inhibited RANKL-induced bone resorption activity in RAW264.7 cells [40]. However, in the present study, GLY extracts did not influence RANKL-induced NFATc1 expression (Figure 3). We did not know the reason for this difference in NFATc1 expression because GLY extracts contain some components. NFATc1 is located in a cytoplasm of osteoclasts as an inactive form that does not have transcriptional activity [8]. When pre-osteoclastic cells are stimulated by RANKL/RANK interaction, NFATc1 in cells translocates from a cytoplasm into a nucleus and exhibits transcriptional activity as an activated form, and then regulates the expression of TRAP, DC-STAMP and cathepsin K, etc. [8,41]. An activation of NFATc1 induced a differentiation of osteoclast precursor to mature osteoclasts, and inhibition of NFATc1 activity resulted in suppressing osteoclast differentiation [36]. Wogonin, a component in GLY formula, inhibited osteoclast differentiation through the inhibition of NFATc1 translocation from the cytoplasm to the nucleus and the downregulation of genes associated with osteoclast differentiation [42]. Since GLY extracts did not suppress RANKL-induced NFATc1 production, but inhibited cathepsin K and DC-STAMP expression and osteoclast differentiation in RAW264.7 cells, GLY extracts might suppress NFATc1 activity by inhibiting the translocation of NFATc1 to a nucleus, but not NFATc1 production. Both our research and Mao et al. [43] found that ephrin B2 was expressed during RANKL-induced osteoclast differentiation in RAW264.7 cells. Ephrin B2 is identified as a target of NFATc1, and its expression is dependent on RANKL-induced NFATc1 transcription during osteoclast differentiation [44,45]. We speculate that a translocation of NFATc1 to a nucleus, but not NFATc1 production, may be suppressed by GLY extracts, since ephrin B2 expression was also down-regulated by GLY extracts in RANKL-induced RAW264.7 cells.

In the present study, GLY extracts showed an inhibitory effect on the ligature-induced alveolar bone resorption by an oral administration for 20 days (Figure 4), suggesting that GLY extracts have a pharmacological effect on a pathological stimulation to periodontal tissues. It was reported that oral administration of Bu-Shen-Gu-Chi-Wan, a traditional Chinese medicine, for four weeks is detected significant changes in alveolar bone volume and density in experimental periodontitis by micro-CT analysis in spite of no improvement of alveolar bone height by stereomicroscopy [46]. It was also reported that the intra-gastric administration of curcumin, an active ingredient of turmeric, for 30 days decrease the alveolar bone resorption in experimental periodontitis [47]. The inhibitory effect of GLY extracts on alveolar bone resorption showed even after a shorter administration period than that of Bu-Shen-Gu-Chi-Wan and curcumin, suggesting that GLY extracts an effective candidate to prevent alveolar bone resorption in periodontitis.

GLY extracts show not only inhibitory effect of bone resorption, but also anti-inflammatory action, and has been prescribed for treatments of stomatitis and glossodynia, as well as periodontitis. GLY extracts contain some components with anti-inflammatory action on some cells and tissues. Baicalin suppressed *P. gingivalis* LPS-induced IL-6 and IL-8 expressions in human oral keratinocytes [48], and baicalin, baicalein, and wogonin inhibited high glucose-induced inflammatory responses, including vascular permeability, expression of cell adhesion molecules, and production of reactive oxygen species (ROS) in HUVECs [49]. Liquiritigenin suppressed the productions of IL-1β, IL-6, and TNF-α when RAW264.7 cells were stimulated by LPS and carrageenan-induced paw edema in rats [50]. Naringin down-regulated the expressions of IL-1β, IL-6, and TNF-α and up-regulated the productions of anti-oxidants, such as glutathione, superoxide dismutase, and catalase in retinal tissues of diabetic rats [51]. Furthermore, GLY extracts suppressed TNF-α level in oral carcinoma cells [20]. The ligature stimulation caused infiltration of inflammatory cells in periodontal tissues of rat experimental periodontitis, and calcitonin, a calcium regulatory hormone, decreased the number of inflammatory cells and the ligature-induced alveolar bone resorption [11]. The relationships between inflammatory responses and bone resorption, osteoclast formation are generally known in periodontitis [52]. Although we did not evaluate the effect of GLY extracts on inflammatory responses in rat periodontal tissues in this study, we suggest that GLY extracts suppressed osteoclast differentiation and alveolar bone resorption by inhibiting inflammatory responses in periodontal tissues.

NTx-1 levels in sera from the rats administered GLY extracts was significantly lower than that of the control (non-administration), whereas osteocalcin level did not change between the administration groups and control (Figure 6A,B). NTx-1 and osteocalcin are biomarkers of bone resorption by osteoclasts and bone formation by osteoblasts, respectively [37]. These results suggest the possibility that GLY extracts systemically influenced the osteoclast activity, but not the osteoblast activity. However, GLY extracts did not show effects on the body weight, and bone volume fraction (BV/TV), bone mineral contents (BMC), and bone mineral density (BMD) in rat femurs used in this study (Figure 7). Thus, the side-effects of GLY extracts on systemic bone metabolism did not occur during the experimental period in this study. We do not know the exact reason why bone quality in the femur does not change in spite of a decrease in serum NTx-1 level. GLY extracts appear to inhibit the osteoclast activity at inflamed periodontal sites, but not systemically influence. But a longer period of administration and observation is needed to investigate systemic effects definitely.

This study suggests the possibility that herbal medicine that internally applies, but not locally administration, prevents alveolar bone resorption in periodontitis. Periodontitis is caused by infection of periodontopathic bacteria and decline of immunological functions in the human host, and results in periodontal tissue degradation. Plaque control, scaling of dental calculus, root planing, curettage of infectious materials, and inflammatory tissues are usually performed for treatments, and prevention of periodontal diseases. Although a few drugs, such as antibiotics and anti-inflammatory agents, are locally and systemically used to suppress inflammation of periodontal tissues, there are few medicines that targeted the prevention of alveolar bone resorption in periodontitis. Gokhale et al. [53] proposed that “systemic host modulatory agents”, such as anti-cytokine agents, inhibitors of mitogen-activated protein kinase (MAPK), and NF-κB, and nitric oxide synthase inhibitors may be useful for periodontal therapies in combination with conventional periodontal treatment. By doing a human study of the combination of conventional periodontal treatment and the administration of GLY extracts, we hope that GLY extracts may become one of the host modulatory agents for the prevention and therapy of periodontitis to encourage better clinical outcomes. To our knowledge, we are the first group to demonstrate a predominant inhibitory function of GLY extracts on bone resorption in periodontitis model rats. We also hope that GLY extracts become an efficacious therapeutic agent to prevent bone destruction in various bone diseases and think that further basic and clinical studies will be necessary to evidence the usefulness of GLY extracts as a medical agent for periodontitis and other bone diseases.

## 5. Conclusions

The present study showed that GLY extracts significantly inhibited sRANKL-induced osteoclast differentiation and decreased the expressions of osteoclast differentiation markers in vitro, and that the administration of GLY extracts for rat experimental periodontitis inhibited the ligature-induced alveolar bone resorption and significantly decreased the number of TRAP-positive osteoclasts around a ligature in the alveolar bone in vivo. We conclude that GLY extracts suppress the alveolar bone resorption by inhibiting the osteoclast differentiation in experimental periodontitis, suggesting that GLY extracts are potentially useful for oral care to suppress the alveolar bone resorption in periodontitis. Further studies with the involvement of a long-term administration of GLY extracts would be beneficial in evaluating the effect of GLY extracts on bone metabolism in periodontitis.

## Supplementary Materials

The following are available online at https://www.mdpi.com/2077-0383/10/3/386/s1, Figure S1: Effects of high concentration of GLY extracts on the RANKL-induced osteoclast differentiation. Figure S2: Effect of GLY extracts on osteoclast differentiation in palatal side of ligature-induced periodontitis.
